# P-1586. Urine Culture Reflex Implemented to Reduce Inappropriate Testing

**DOI:** 10.1093/ofid/ofae631.1753

**Published:** 2025-01-29

**Authors:** Erika Orner, Adam Zimilover, Sammy Cheng, Kelsie Cowman, Phyu Thwe, Wendy Szymczak, Inessa Gendlina

**Affiliations:** Montefiore Medical Center, Bronx, New York; Montefiore Medical Center - Albert Einstein College of Medicine, Bronx, New York; Albert Einstein College of Medicine, Bronx, New York; Montefiore Medical Center, Bronx, New York; Montefiore Medical Center, Bronx, New York; Montefiore Medical Center, Albert Einstein College of Medicine, Bronx, NY; Albert Einstein College of Medicine, Bronx, New York

## Abstract

**Background:**

Improper ordering of urine cultures and patient care based solely on positive culture in the absence of urinalysis suggestive of infection can lead to overtreatment of asymptomatic bacteriuria, increased healthcare costs, and increased risk of antimicrobial-related adverse events like *C. difficile* infection and increased antimicrobial resistance.

Table 1

**Figure:**
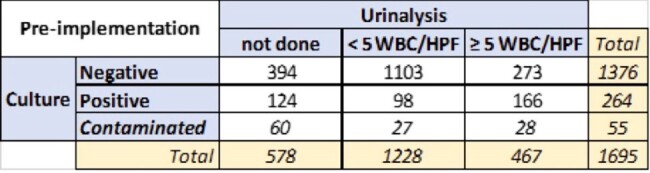

Results of specimens collected Oct 24-Nov 4, 2022 pre-implementation

**Methods:**

A reflex urinalysis-to-urine culture was implemented with a threshold of urine culture performed only when 5+ WBC/HPF are seen in urinalysis. Pre-implementation (10/24/22–11/4/22) data from inpatients with concurrent urinalysis and urine culture was analyzed to determine how results would be impacted if urinalysis was utilized as criteria to perform urine culture. Post-implementation (10/24/23–11/4/23) data was also analyzed.

Table 2

**Figure:**
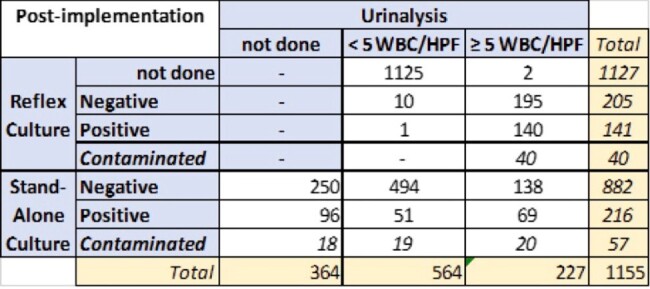

Results of specimens collected Oct 24-Nov 4, 2023 post-implementation

**Results:**

Pre-implementation, 1428 urine cultures were done on average per week. During the pre-implementation period, 1695/2273 patients had UA done on the same day as culture. 1376/1695 cultures were negative, 264/1695 cultures were positive, and 55/1695 cultures were contaminated ( ≥3 organisms). Using 5+ WBC/HPF as culture criteria, 1228/1695 (72%) specimens would not have qualified for culture and only 5.8% of cultures would have been falsely negative. Post-implementation, there were 863 urine cultures/week on average. 1125/1513 reflex specimen did not qualify for culture. 375/1513 had 5+ WBC/HPF and qualified for culture but only 140/377 were culture positive. 791/1155 standalone cultures had UA done on the same day. Only 227/791 would have qualified for culture.

Figure 1

**Figure:**
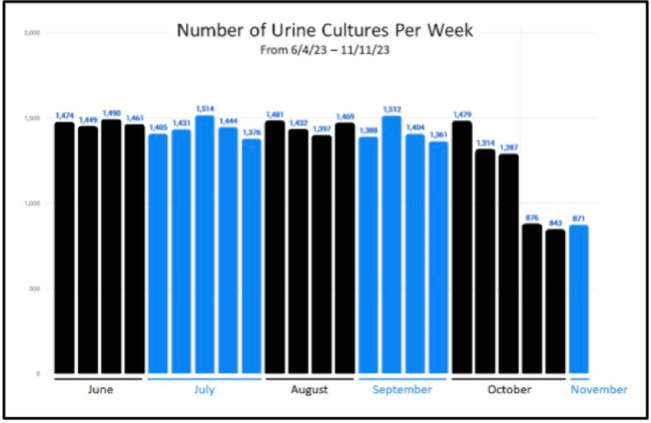

Number of urine cultures performed weekly from June to November 2023. Implementation of reflex testing was done Oct 24, 2023

**Conclusion:**

As predicted, using WBCs as criteria to perform urine culture has significantly reduced our urine culture volume. 14% of cultures are still being done without corresponding UA indicating test utilization could further improve. Future studies will analyze the impact reflex testing has on clinical diagnosis and treatment.

**Disclosures:**

**Wendy Szymczak, PhD**, Quidel: Advisor/Consultant

